# Single-step generation of metal-plasma polymer multicore@shell nanoparticles from the gas phase

**DOI:** 10.1038/s41598-017-08274-6

**Published:** 2017-08-17

**Authors:** Pavel Solař, Oleksandr Polonskyi, Ansgar Olbricht, Alexander Hinz, Artem Shelemin, Ondřej Kylián, Andrei Choukourov, Franz Faupel, Hynek Biederman

**Affiliations:** 1 Charles University, Faculty of Mathematics and Physics, Department of Macromolecular Physics, Prague, 182 00 Czech Republic; 2 Kiel University, Faculty of Engineering, Chair for Multicomponent Materials, 24143 Kiel, Germany

## Abstract

Nanoparticles composed of multiple silver cores and a plasma polymer shell (multicore@shell) were prepared in a single step with a gas aggregation cluster source operating with Ar/hexamethyldisiloxane mixtures and optionally oxygen. The size distribution of the metal inclusions as well as the chemical composition and the thickness of the shells were found to be controlled by the composition of the working gas mixture. Shell matrices ranging from organosilicon plasma polymer to nearly stoichiometric SiO_2_ were obtained. The method allows facile fabrication of multicore@shell nanoparticles with tailored functional properties, as demonstrated here with the optical response.

## Introduction

Production of metal nanoparticles (NP) by gas aggregation sources (GAS) that utilize magnetron sputtering and buffer-gas condensation has become a fast-growing field in nanoscience^1–5^. Sputtering can supply atomic metal vapors from solid targets into a gas phase in a controllable manner. By adjusting the parameters of the discharge, the partial pressure of metal vapors can be deliberately increased above the target surface to create the conditions of supersaturation. Such far-from-equilibrium conditions can be further enhanced by using a cool buffer gas which may trigger spontaneous condensation of metal vapors and the formation of NPs. Typically, a buffer gas flow is created in the aggregation zone to transport NPs away from the magnetron and deposit them onto solid supports.

Originating in the 1990s, work on the use of magnetron-based GASes for the production of single-metal NPs^6–9^ was followed by research on multi-metal NPs. The combination of two (or more) metals at the nanoscale was found to provide NPs with unique properties and extra functionality. The state-of-the-art involves using a single magnetron with a composite target or multiple magnetrons in a single GAS. It was recognized that multi-metal NPs of different morphology can be produced, for example, nanoalloy^10–13^, core@shell^2, 14–18^ or Janus NPs^19, 20^.

Fabrication of composite NPs composed of materials other than metals can significantly broaden the functionality of NPs. Possible applications include protection of metal NPs from oxidation, fine tuning NP plasmonic response and functionalization of NPs for controlled drug delivery and biosensing. Composite NPs with metal core and dielectric shell were successfully produced using wet chemical methods^21–23^ or combinations of physical and chemical methods^24^. These, however, usually need multiple production steps and typically suffer from the use of toxic precursors, the elimination of the residuals of which represents a substantial challenge. From this viewpoint, gas phase methods of fabrication of composite NPs may prove advantageous albeit they have been studied much less^25^.

In this paper, we present a method for the production of metal core@plasma polymer shell NPs that combines buffer-gas induced condensation of magnetron-sputtered silver and plasma-enhanced chemical vapor deposition (PECVD) of hexamethyldisiloxane (HMDSO). The aim of the research is to investigate the feasibility of such an approach and to find tools to tune the morphology and chemical composition of NPs by setting the appropriate experimental conditions.

## Experimental

**Figure 1:**
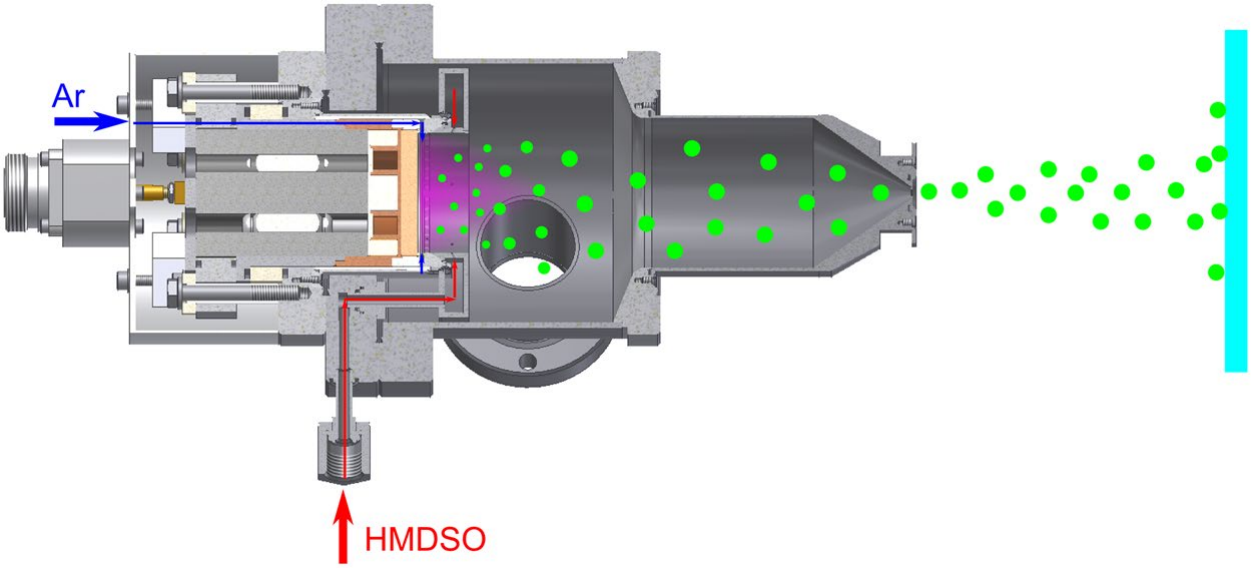
Schematics of the gas aggregation cluster source and the gas inlet system.

Nanoparticles (NPs) were produced using a gas aggregation cluster source (GAS) with a 2-inch magnetron equipped with either a graphite or a silver target (both 6 mm thick, 99.99% pure, Kurt J. Lesker). The magnetron was placed inside a cylindrical aggregation chamber capped with a conical lid with a circular orifice 3 mm in diameter and 0.5 mm in length. The GAS chamber was cooled by water at 20 °C. The magnetron was powered by an RF generator (Dressler Cesar, 13.56 MHz) through a matching box. Constant power of 50 W was chosen for the experiments. Argon (99.9999%) was supplied through a flow controller (MKS Instruments) between the powered electrode and the magnetron shielding directly to the target, as shown in Fig. 1. Hexamethyldisiloxane (HMDSO, 98.5% pure, Sigma Aldrich) was supplied to the GAS from a flask via a needle valve. The HMDSO inlet ends in a gas ring positioned 15 mm away from the surface of the target. The gas ring had several holes, all directed towards the target. The experiments were performed at 190 Pa pressure in the GAS with the flow rate of Ar fixed at 105 sccm. The concentration of HMDSO was varied by changing its flow rate from 0.02 to 0.45 sccm. In a number of the experiments, O_2_ (99.9999%) was added to the Ar/HMDSO mixture.

The GAS was mounted onto a high vacuum deposition chamber pumped by scroll and turbo-molecular pumps to a base pressure below 10^–4^ Pa. The NPs were deposited on different substrates (quartz glass, TEM grids, gold coated silicon substrates) positioned in the deposition chamber 20 cm from the exit orifice of the GAS. The samples were analyzed by transmission electron microscopy (TEM, JEM-2100, JEOL, 200 kV, LaB6), UV-Vis spectroscopy (Ellipsometer Woollam M2000 UI), X-ray photoelectron spectroscopy (XPS, Omicron Nanotechnology GmbH) and Fourier Transform Infrared Spectroscopy (FTIR, Bruker Equinox 55). In the case of XPS measurements, all spectra were charge referenced for aliphatic carbon at 285.0 eV.

## Results and Discussion

**Figure 2:**
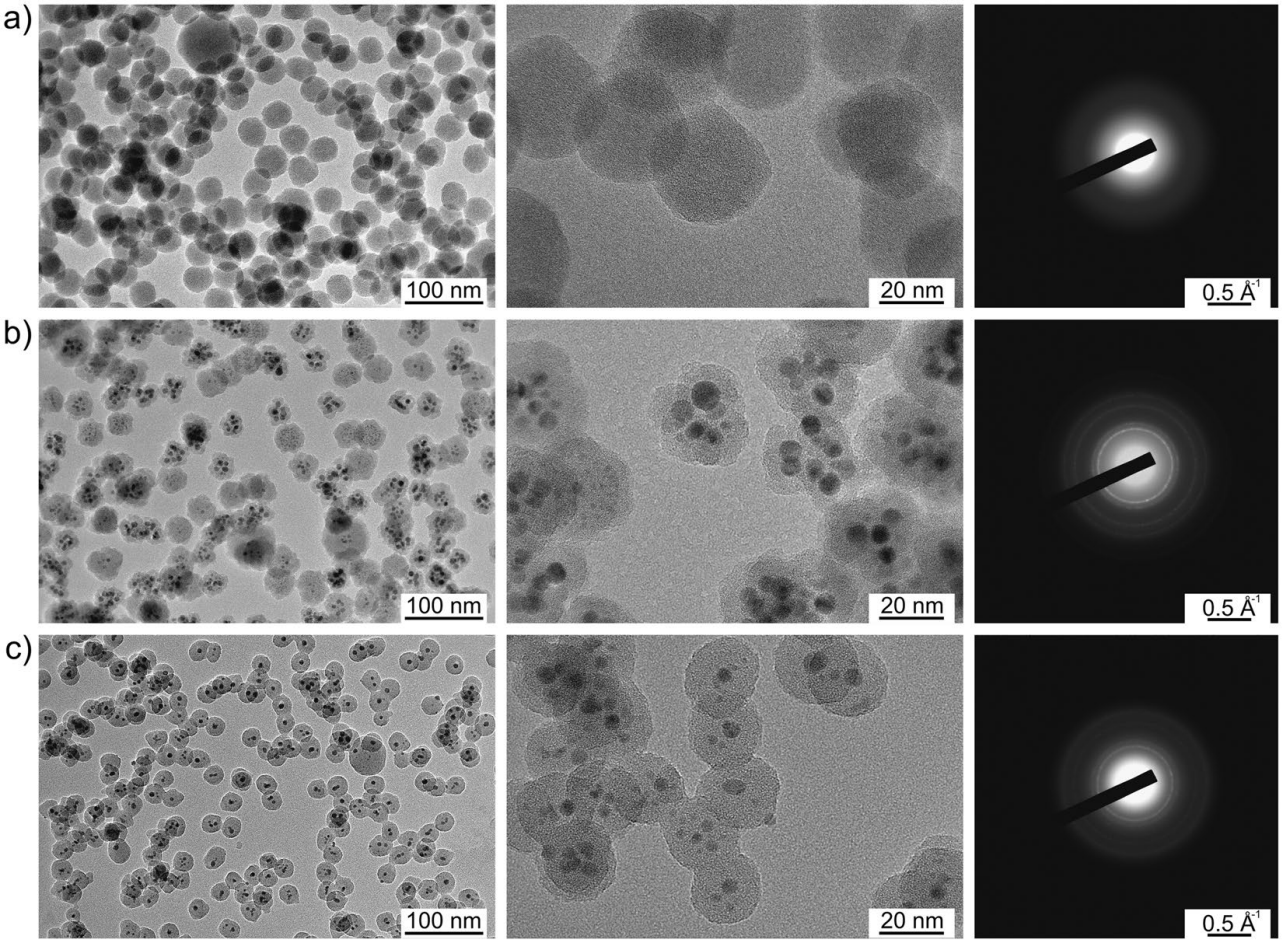
TEM images with lower (left column) and higher (middle column) magnification of the NPs prepared by GAS: (**a**) from the Ar/HMDSO mixture with graphite target and (**b**) from the Ar/HMDSO mixture with Ag target (in both cases the HMDSO flow rate is 0.45 sccm); (**c**) from the Ar/HMDSO/O_2_ mixture with Ag target (0.15% HMDSO, 3.8% O_2_, Ar flow rate 105sccm, pressure 190 Pa). The right column shows the corresponding diffraction patterns.

Plasma polymerization of organic precursors often leads to the formation of nano- and micro-scale particles in the gas phase. Organic molecules supplied to the plasma zone undergo fragmentation followed by gas-phase radical recombination and form nuclei, the process being especially effective if performed under an elevated pressure of tens to hundreds of Pa^26^. Chemical composition of the resultant particles is given by the type of precursor used and by the energetic conditions of the plasma. Particle structure can typically be represented by a highly cross-linked and random carbonaceous network^27^. Figure 2a shows an example of the NPs prepared by the GAS with a mixture of Ar and HMDSO and a graphite target attached to the magnetron. The NPs were produced with a mean size of 36 nm and with the XPS elemental composition of C 52%, Si 30%, O 18%, very similar to the HMDSO films and NPs reported earlier^28, 29^. The TEM image in Fig. 2b shows the result of the same experiment performed when the graphite target was replaced by a silver one. In this case, heterogeneous NPs are produced and consist of distinct multiple cores enveloped by a shell of the plasma polymer. XPS analysis shows the appearance of silver, although the concentration of other elements remains almost the same (Ag 2%, C 52%, Si 25%, O 21%). Remarkably, the mean size of the multicore@shell NPs does not change when compared to the NPs without the multicores. The diffraction pattern characteristic for silver was detected by TEM and is shown in the right column of Fig. 2.

Magnetron sputtering of metals is known to result in the supply of an atomic metal vapor into the gas phase. The metal vapor may spontaneously condense with the formation of NPs if the conditions of supersaturation are fulfilled, and recent years have witnessed the successful application of GASes for the production of beams of metal NPs^8, 30, 31^. Here, the magnetron sputtering of silver is evidently accompanied by plasma polymerization processes of HMDSO-derived radicals and the formation of Ag NPs is accompanied by competitive growth of HMDSO plasma polymer. It is known that cohesive forces between noble metal atoms greatly exceed the interaction energy between metals and organics, polymers in particular. As a consequence, metals do not form one phase with polymers but both tend to segregate into two phases^32^. Within the framework of low-temperature plasma deposition, the phase segregation leads to the generation of metal/plasma polymer nanocomposites, either in the common form of thin films^33–36^ or in the form of core@shell NPs presented here. It is also worth noting that the growth does not lead to the formation of individual core@shell NPs, as might be and was originally expected, but the groups of several Ag NPs become embedded in a single plasma polymer shell. It seems reasonable to assume that individual core@shell NPs are formed in the vicinity of the magnetron target and later coalesce with each other on their way through the aggregation chamber, thus generating the multicore@shell structure. A similar phenomenon was recently observed for ternary magnetron synthesis of inorganic NPs composed of multiple FeAg cores encapsulated by a Si shell^37, 38^.

**Figure 3:**
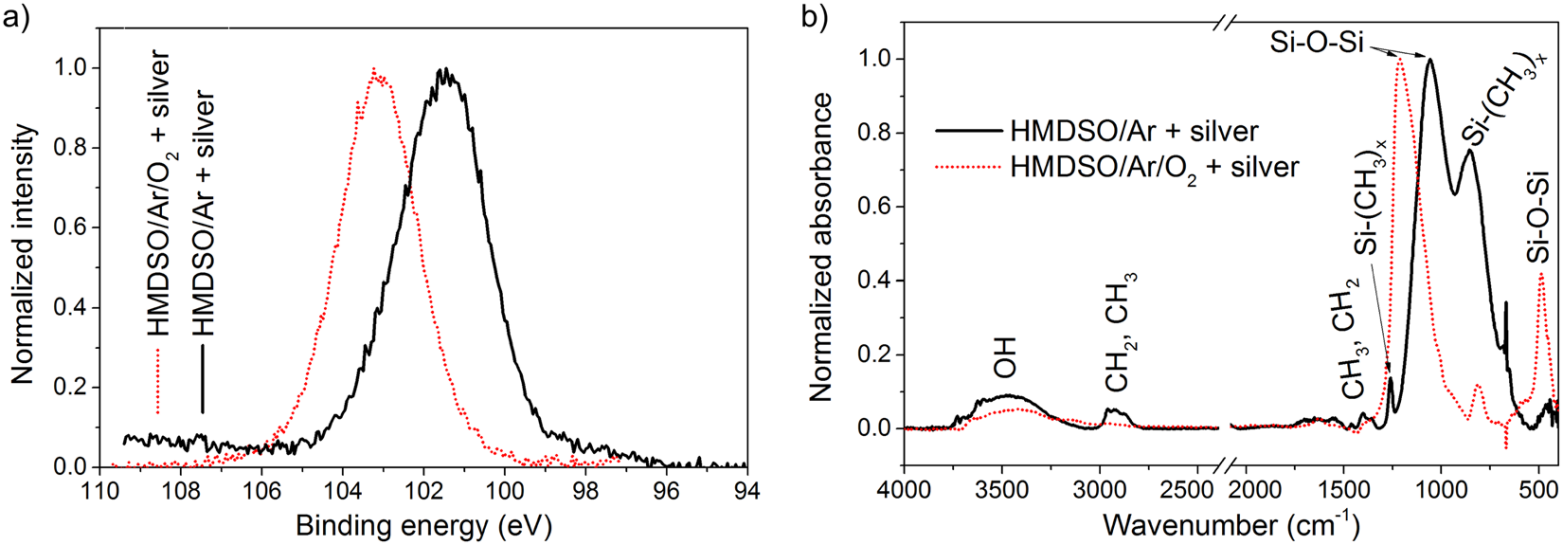
Chemical analysis of the NPs prepared by GAS from the Ar/HMDSO mixture with or without the addition of O_2_: (**a**) high-resolution Si 2p XPS; (**b**) FTIR-RAS.

Addition of oxygen to HMDSO is known to trigger oxidation processes in the plasma. Reactive oxygen species consume carbon with the formation of volatile oxides that do not participate in plasma polymerization. This in turn leads to the production of carbon-deficient deposits that are consequently more inorganic. The process can be optimized to produce stoichiometric SiO_2_ in the form of thin films or nanoparticles^29^. Figure 2c shows the result of such optimization performed in the GAS with the silver target. The addition of 4% of O_2_ to the Ar/HMDSO mixture leads to the fabrication of NPs with embedded multiple Ag inclusions with 5 nm average size. The XPS composition shows Ag 4%, C 3%, Si 32%, O 61%, which indicates that the shell consists of nearly stoichiometric SiO_2_. No diffraction pattern characteristic of crystalline silicon dioxide was detected, which points to an amorphous state of the shell. The formation of SiO_2_ is also corroborated by high-resolution XPS, which shows a higher binding energy shift in the position of the Si 2p peak from 101.6 eV for the Ar/HMDSO mixture without O_2_ to 103.1 eV for the mixture with O_2_ (Fig. 3a). Furthermore, the FTIR-RAS spectra of both types of NPs also reveal the chemical changes from the organic to the predominantly inorganic SiO_2_ character (Fig. 3b). The addition of O_2_ leads to the disappearance of the bands associated with vibrations of the hydrocarbon and organosilicon groups (see, for example, CH_x_ stretching at 2960–2870 cm^−1^ and Si-(CH_3_)_x_ rocking at 850 cm^−1^). Simultaneously, a siloxane rocking band develops at 485 cm^−1^, whereas the corresponding stretching vibration shifts from 1050 to 1210 cm^−1^, which also reflects the disappearance of an organic bonding environment^33, 39^.

**Figure 4:**
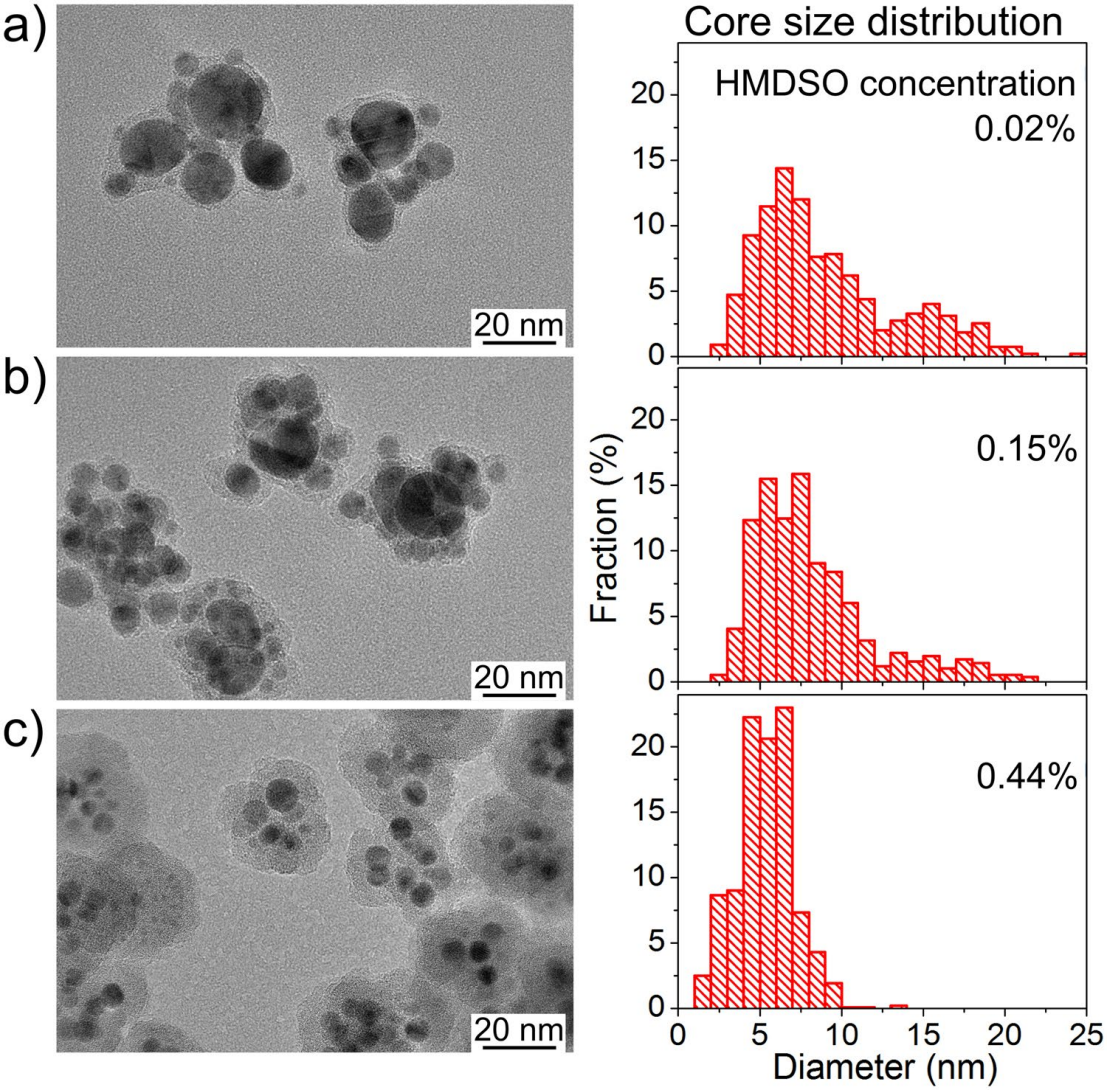
TEM images and the core size distributions of the multicore-shell NPs prepared by GAS at different concentrations of HMDSO: (**a**) 0.02%, (**b**) 0.15%, (**c**) 0.44%.

Another set of experiments was performed without the addition of O_2_ but with different concentrations of HMDSO in Ar. All other parameters were held constant. The size of the Ag inclusions was found to decrease and the thickness of the plasma polymer envelope to increase, with the HMDSO concentration increasing from 0.02% to 0.44% (Fig. 4). The phenomenon is accompanied by the narrowing of the Ag NP size distribution, as can be seen from the corresponding histograms. The XPS analysis supports these findings by showing the decrease of the silver content from 27 at. % to 2 at. % with the increasing thickness of the plasma polymer shell (increasing concentration of HMDSO).

**Figure 5:**
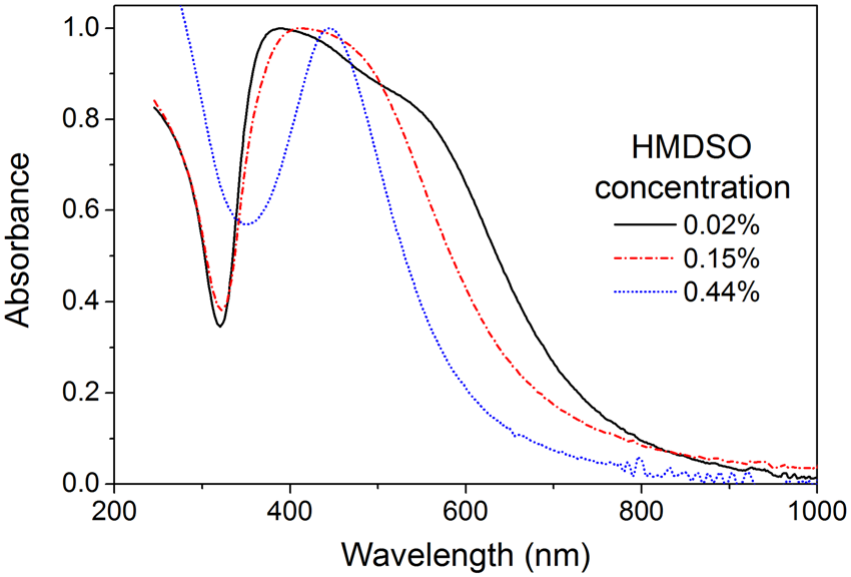
UV-Vis spectra of the core-shell nanoparticles prepared by GAS at different concentrations of HMDSO.

The UV-Vis measurements of the multicore@shell NPs provide spectra with a strong absorption band from localized surface plasmon resonance (LSPR) (Fig. 5). At the lowest HMDSO concentration, the band occupies a region between 400 and 600 nm, a large width of which is caused by the large dispersity in Ag NP size. Also, note the presence of two shoulders in this band that likely originate from the bimodal size distribution of the Ag NPs detected in the sample (Fig. 4). In further agreement with the TEM observations, the increase of HMDSO concentration leads to a narrowing of the LSPR absorption band, and corresponds to a narrower Ag NP size distribution. The position of the band at 445 nm is red-shifted with respect to <400 nm typically obtained for similarly sized bare Ag NPs^31^. The red-shift of the LSPR band has been well documented in the literature, the shift being larger for embedding dielectric media with greater refractive index^40^. Here, the shift is readily attributed to the influence of the HMDSO plasma polymer matrix separating the Ag NPs as compared to bare Ag NPs separated by air voids. It also confirms that the Ag NPs are completely embedded within the plasma polymer shell.

## Conclusion

An effective single-step method for the gas-phase production of metal@plasma polymer nanoparticles was developed and is based on the use of a gas aggregation cluster source for simultaneous rf magnetron sputtering of a metal target and PECVD of an organic precursor. Silver NPs were successfully embedded in an organosilicon plasma polymer shell. Advantageously, the method also involves the formation of multicore@shell instead of a single-core@shell morphology of NPs. The concentration of HMDSO is found to be an important parameter influencing the size distribution of the Ag inclusions as well as the optical activity of the resultant multicore@shell NPs. Adding oxygen to the Ar/HMDSO mixture permits tuning of the chemical composition of the shell from organosilicon plasma polymer to stoichiometric SiO_2_. The facile, single-step method presented here should lend itself to the controlled fabrication of other complex functional nanoparticles.
